# Diagnostic value of plasma cell-free DNA metagenomic next-generation sequencing in patients with suspected infections and exploration of clinical scenarios—a retrospective study from a single center

**DOI:** 10.1080/07853890.2025.2608531

**Published:** 2026-01-02

**Authors:** Shuhong Sun, Zhen Ning, Hongyan Xu, Shanxin Peng, Chunhai Gao, Xiaofeng Hu

**Affiliations:** ^a^ Department of Laboratory Medicine, Shandong Provincial Linyi Peoples’ Hospital, Shandong Provincial Key Medical and Health Laboratory, Shandong Provincial Linyi Municipal Key Laboratory for Laboratory Medicine, Linyi, Shandong, China; ^b^ Department of Hospital Infection Management, Shandong Provincial Linyi Peoples’ Hospital, Shandong Provincial Key Medical and Health Laboratory, Shandong Provincial Linyi Municipal Key Laboratory for Laboratory Medicine, Linyi, Shandong, China

**Keywords:** Plasma cell-free DNA, metagenomic next-generation sequencing, infectious diseases, clinical impact

## Abstract

**Background:**

Plasma cell-free DNA metagenomic next-generation sequencing (mNGS) is a non-invasive comprehensive method for the etiological diagnosis of various infectious diseases. However, research on the early diagnosis and real-world clinical impact of plasma mNGS in patients with suspected infection are still limited.

**Materials and Methods:**

This study retrospectively included 140 patients with suspected infections who underwent early plasma mNGS and conventional culture testing. Referring to the clinical diagnosis of infectious diseases, the diagnostic performance of plasma mNGS and culture tests was compared, and the application scenarios and clinical effects of plasma mNGS were evaluated.

**Results:**

The positive rate of plasma mNGS was significantly higher than that of culture methods (55.71% vs 25.10%, *p* < 0.001) and blood cultures (55.71% vs 12.86%, *p* < 0.001). Regarding clinical diagnosis, the sensitivity of plasma mNGS was significantly higher than that of culture (58.27% vs 37.80%, *p* = 0.002). The combination of mNGS and culture achieved a higher detection sensitivity (69.29%), especially in patients with multi-site co-infections (73.68%) and blood infections (73.17%). Plasma mNGS demonstrated higher sensitivity in patients with procalcitonin (PCT) index > 5 ng/ml or human neutrophil lipocalin (HNL) index > 200 ng/ml. In terms of treatment, a total of 69 patients (54.33%) benefited from plasma mNGS.

**Conclusion:**

This study highlights the significant improvement in pathogen detection performance by combining conventional culture with plasma mNGS detection, especially in patients with multi-site co-infections and blood infections. Early use of plasma mNGS as an adjunct to culture can better guide clinicians to initiate appropriate anti-infective therapy.

## Introduction

Infectious diseases are the leading cause of morbidity and mortality worldwide and present a rising threat to public safety [1,2]. Timely and accurate identification of the cause of infection is essential for targeted therapy [3]. Despite the increasing sensitivity of detection techniques, pathogen identification of infectious diseases remains a critical issue for clinicians [4].

At present, clinical laboratory departments usually detect pathogens through smear microscopy, culture, serological tests, pathological examinations, and nucleic acid amplification tests, however, the sensitivity and detection ability of these methods have limitations [5]. Conventional microbial cultures and smear microscopy remain the gold standard for diagnosis. However, these methods are time-sensitive, especially for slow-growing microorganisms, and do not effectively culture uncultivatable or fastidious pathogens [6]. Conventional microbiological test methods are not sufficient to meet clinical needs, especially in patients with suspected infections and unknown etiologies [7]. Some markers of infection and inflammatory responses, such as serum procalcitonin (PCT), C-reactive protein (CRP), interleukin-6 (IL-6), and human neutrophil lipocalin (HNL), have been tested for their ability to distinguish between infectious and non-infectious, but the etiology of infection has still not been demonstrated [3,8].

Metagenomic next-generation sequencing (mNGS) has been widely studied in a variety of infectious diseases. mNGS does not rely on culture and can detect all DNA without bias, making it particularly useful for rare and atypical etiologies of complicated infectious diseases [4,9]. Clinicians want to quickly determine the cause of suspected infections in patients, which can reduce the need for empiric therapy. Blood is widely used as a non-invasive sample to detecting infections. Zhang et al. also demonstrated the importance of mNGS detection using plasma cell-free DNA (cfDNA) in patients with early infection [3]. However, due to uncertainty about the site of infection, only a few positive results can be obtained from blood samples [10]. The applicability and clinical impact of plasma testing are poorly understood in different populations of patients. So far, mNGS studies of blood samples have been applied to a variety of diseases, including bloodstream infections, sepsis, febrile neutropenia, and infective endocarditis, which have demonstrated robust detection performance [11–14]. However, in the early stages of infection, the cause of infection in these patients is not clear, and timely exclusion of infection or targeted therapy is extremely important for the prognosis of patients. Only a few studies have focused on the use of plasma mNGS in patients with early suspected infections, and the clinical impact of mNGS remains unclear. Herein, 140 patients who underwent plasma mNGS and cultures to retrospectively evaluate the diagnostic performance and clinical impact of these two detection methods, based on their final clinical diagnosis of infectious diseases.

## Materials and methods

### Patient enrolment and study design

The clinical data of 167 patients with suspected infections who were hospitalized at Linyi People’s Hospital in Shandong Province between February 2022 and December 2023 and underwent early plasma mNGS testing were retrospectively collected. Inclusion criteria for the study: Patients with high suspicion of infectious clinical manifestations. Exclusion Criteria: (1) incomplete cases; (2) The cultures were not detected simultaneously with mNGS; (3) patients identified as having non-infectious diseases prior to mNGS testing.

This study was approved by the Ethics Committee of Linyi People’s Hospital, Shandong Province, China (202411-H-001). All procedures involving human participants were in accordance with the Declaration of Helsinki.

Blood samples from 140 patients were collected for plasma mNGS testing and blood cultures. Samples from multiple suspected sites of infection from each patient were used for culture, including sputum, urine, cerebrospinal fluid (CSF), secretions, tissue, bronchoalveolar lavage fluid (BALF), and pus.

### Clinical data collection

Clinical data collected for these patients included demographic data, comorbidities, sites of infection, clinical indicators (such as white blood cell count (WBC), percentage of neutrophils to total white blood cells (NE%), procalcitonin (PCT) and C-reactive protein (CRP) and Human neutrophil lipocalin (HNL) levels), length of hospital stay (days), clinical diagnosis, and outcome at discharge.

### mNGS sequencing and analysis

Blood samples were collected into anticoagulant tubes and subsequently cryotransported to Willingmed Co., Ltd. (Beijing, China) for mNGS. Plasma isolation, nucleic acid extraction, and library preparation were performed as previously described [15]. For nucleic acid extraction, the whole blood samples were centrifuged at 1900 g at 4 °C for 10 min, and the upper plasma were collected for subsequent processing. The pooled libraries were sequenced on the NextSeq^™^ 550Dx system using a 75 bp, single-end sequencing kit (Illumina, San Diego, USA), ensuring a minimum of 20 million sequencing reads per sample [16]. Sequencing data in raw FASTQ format was quarantined and evaluated using Trimmomatic v0.40, filtering for low-quality or undetected sequences, high-coverage repeats, short-read-length sequences, and more to preserve high-quality sequencing data [17]. Bowtie2 v2.4.3 was then aligned to the human reference genome GRCh37 (hg19) to remove the human sequence [18]. The acquired data were compared with the non-redundant nucleotide sequence database of the National Center for Biotechnology Information (NCBI) in United States using Kraken2 v2.1.0 to complete the annotation of pathogenic microbial species and give the final microbial analysis and identification results [19].

To identify pathogens, we use RPTM (reads per ten million) to identify positive pathogens. Bacteria and fungi with RPTM ≥ 8 [20], viruses with RPTM ≥ 3, and special pathogens (including Cryptococcus, Mycobacterium, Mycoplasma, Chlamydia, Legionella, and parasites) with RPTM ≥ 1, was identified as ­positive [21–23].

### Evaluation of the clinical impact of plasma mNGS results on patient treatment

The clinical impact of mNGS on patient treatment was retrospectively blinded evaluated by two specialized clinicians with reference to previous grading criteria [15,24]. When there was a disagreement between the two clinicians, the judgment was made based on the result of a third expert’s blinded assessment. There are three levels of effect of mNGS on treatment: positive, no effect, and negative. Positive results indicate that plasma mNGS can contribute to antibiotic adjustment. The absence of an effect indicates that antibiotic adjustment has not been made clinically despite negative or positive plasma mNGS results. A negative result means that unnecessary treatment was carried out based on the results of mNGS [15].

### Statistics analysis

Independent variables were expressed as counts and percentages. The chi-square test was used for the analysis of categorical variables. Continuous variables with a normal distribution were expressed as mean ± standard deviation (mean ± SD), and the significance of differences between the two groups was calculated using the Student t-test. Continuous variables with a non-normal distribution were expressed as median and interquartile range (M, IQR), and the significance of differences between the two groups was determined by the Mann-Whitney U test. Data analysis was performed using SPSS 26.0 software (IBM, United States). A p-value of less than 0.05 were considered statistically significant.

## Results

### Patient characteristics

The clinical and demographic characteristics of the study patients were shown in Table 1. A total of 140 patients were enrolled, of whom 95 (67.86%) were male. The median age of patients was 58 years. Hypertension, diabetes, heart disease, chronic kidney disease, and hyperlipidemia were present in 30.00%, 19.29%, 19.29%, 8.57% and 5.71% of patients, respectively. 20% of the patients were immunosuppressed. Of the 140 patients, 127 (90.71%) were eventually diagnosed with infectious diseases and 13 patients (9.29%) had non-infectious diseases. Based on the final clinical diagnosis, the most common sites of infection identified in infected patients were the lung (66.14%) and blood (64.57%). The PCT index was higher in infected patients than in non-infected patients (1.14 ng/mL vs 0.38 ng/mL, *p* = 0.053).

**Table 1.** Clinical characteristics of participants.

Characteristics	Total (*n* = 140)	Infectious Group (*n* = 127)	Non-infectious Group (*n*= 13)	P value
Age, years (M, IQR)	58 (44.30-71.80)	58 (44.00-71.00)	58 (48.00-72.00)	0.650
Male (n, %)	95 (67.86%)	84 (66.14%)	11 (84.62%)	0.224
Comorbidity (n, %)				
Hypertension	42 (30.00%)	40 (31.50%)	2 (15.38%)	0.344
Diabetes	27 (19.29%)	25 (19.69%)	2 (15.38%)	>0.999
Heart disease	27 (19.29%)	25 (19.69%)	2 (15.38%)	>0.999
Chronic kidney disease	12 (8.57%)	11 (8.66%)	1 (7.69%)	>0.999
Hyperlipidemia	8 (5.71%)	7 (5.51%)	1 (7.69%)	0.551
Immunosuppression (n, %)	28 (20.00%)	26 (20.47%)	2 (15.38%)	>0.999
Hematological disease (n, %)	77 (55.00%)	72 (56.69%)	5 (38.46%)	0.250
Hematological malignancy (n,%)	19 (13.57%)	18 (14.17%)	1 (7.69%)	>0.999
Site of infection (n, %)				
Lung	84 (60.00%)	84 (66.14%)	–	–
Blood	82 (58.57%)	82 (64.57%)	–	–
Abdomen	11 (7.86%)	11 (8.66%)	–	–
Brain	8 (5.71%)	8 (6.30%)	–	–
Urinary tract	8 (5.71%)	8 (6.30%)	–	–
Soft tissue	4 (2.86%)	4 (3.15%)	–	–
Inflammation biomarker				
WBC, (mean ± sd) 10^9/L	11.47 ± 10.70	11.68 ± 10.99	9.42 ± 11.68	0.470
NE%, (mean ± sd)%	75.85 ± 22.64	76.25 ± 23.10	71.94 ± 17.84	0.515
PCT, (M, IQR) ng/mL	0.89 (0.21-12.12)	1.14 (0.24-14.39)	0.38 (0.09-0.89)	0.024
HNL, ng/mL	198.06 ± 132.33	199.15 ± 134.84	176.70 ± 74.11	0.743
CRP, (M, IQR) mg/L	79.68 ± 65.93	77.48 ± 65.95	99.65 ± 65.72	0.315

IQR: interquartile range; WBC: white blood cell; NE%: Percentage of neutrophils in blood; PCT: procalcitonin; CRP: C-reactive protein; HNL: human neutrophil lipocalin.

### Comparison of the diagnostic performance of mNGS and culture

**Figure 1.**
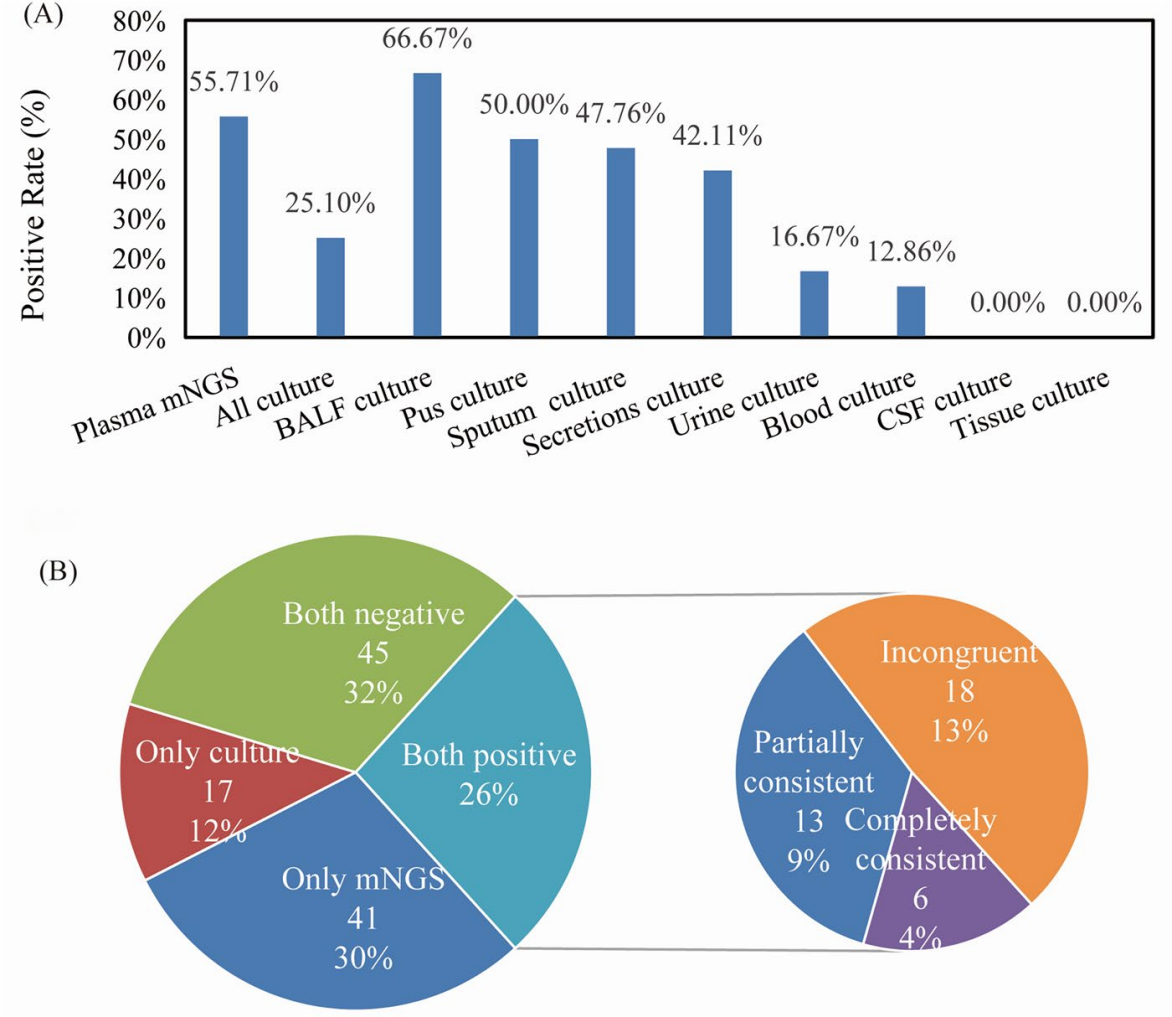
Comparison of diagnostic performance between mNGS and culture. (A) Comparison of mNGS positivity rates with laboratory cultures. (B) Consistency of detection of mNGS and culture.

We compared the positive rates of mNGS and culture methods, as shown in Figure 1A. In addition to the blood samples collected from each patient, 67, 24, 19, 3, and 2 samples were collected from sputum, urine, secretions, BALF and pus, respectively. For all 140 patients, the positive rate of plasma mNGS was 55.71%, which was much higher than that of blood culture (12.86%) (*p* < 0.001). According to the culture results, 66.66% of BALF samples, 50% of pus samples, 47.76% of sputum samples, 42.11% of secretion samples, and 16.67% of urine samples were detected with positive results. As shown in Figure 1B, mNGS and culture were positive in 37 patients and negative in 45 patients. A total of 41 patients were positive for mNGS alone, and 17 patients were positive for culture alone. For double-positive samples, the results were found to be completely consistent for 6 patients, completely inconsistent for 18 patients, and partially consistent for 13 patients, indicating that when a positive result for multiple pathogens is obtained, at least one pathogen overlaps.

The clinical diagnosis of infection is the gold standard. The sensitivity, specificity, positive predictive value (PPV), and negative predictive value (NPV) of mNGS were 58.27%, 76.92%, 96.10%, and 15.87%, respectively (Table 2). The sensitivity, specificity, PPV and NPV of culture were 37.80%, 76.92%, 94.12% and 11.24%, respectively. The sensitivity of mNGS was higher than that of culture (58.27% vs 37.80%, *p* = 0.002). When mNGS was combined with the culture method, the sensitivity of the detection was increased to 69.29%.

**Table 2.** Diagnostic performance of mNGS and culture.

Diagnostic testing	Sensitivity (%)	Specificity (%)	PPV (%)	NPV (%)
mNGS	58.27	76.92	96.10	15.87
Culture	37.80	76.92	94.12	11.24
mNGS + Culture	69.29	61.54	94.62	17.02

PPV: Positive predictive value; NPV: Negative predictive value.

### Pathogens detected by mNGS and culture

**Figure 2.**
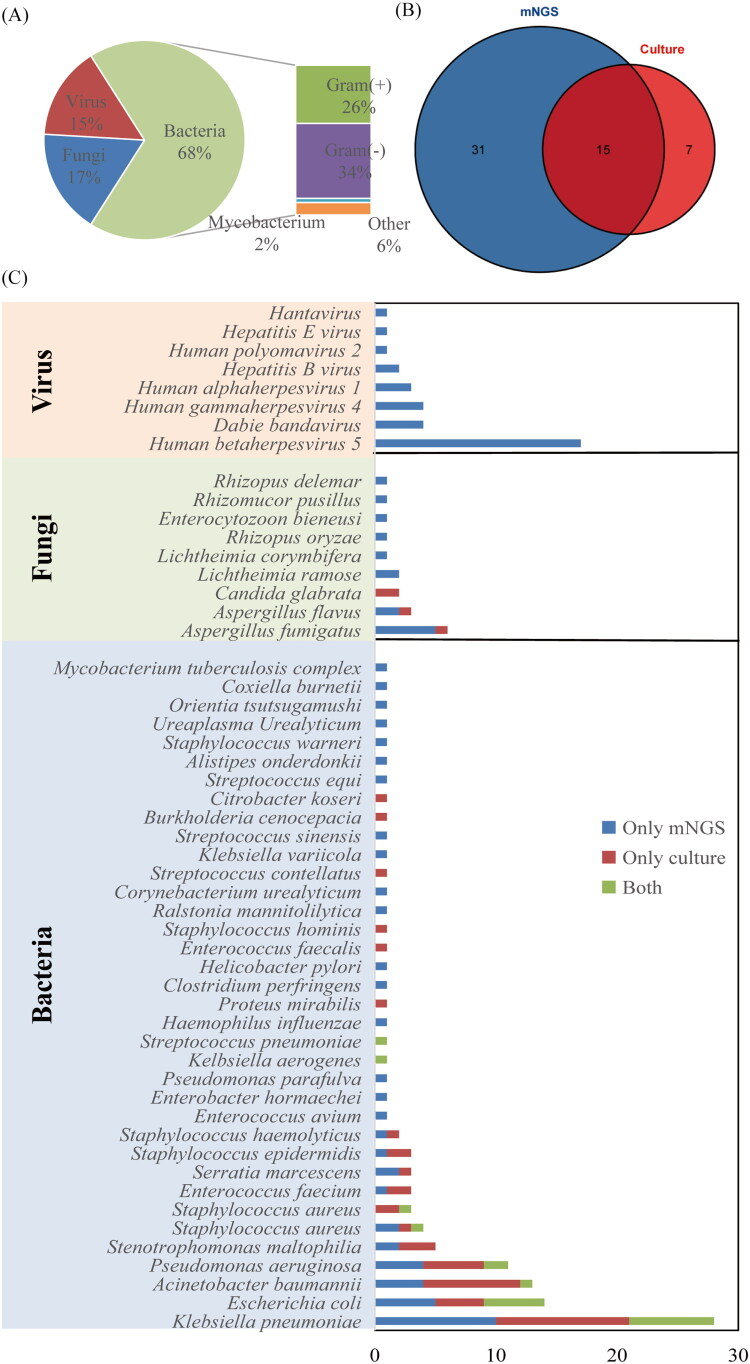
Pathogen spectrum detected by mNGS and culture. (A) Type of pathogen. (B) Venn plot of mNGS and culture of pathogens detected. (C) Distribution of pathogens detected by mNGS and culture.

Among the 140 patients with suspected infection, 53 pathogens, including 36 bacteria, 9 fungi, and 8 viruses, were detected by mNGS and culture methods (Figure 2A). Among them, 31 pathogens were detected by mNGS only, 7 pathogens were detected by culture only, and 15 pathogens could be detected by both mNGS and culture (Figure 2B).

Among the isolated microorganisms, the most common bacteria detected were *Klebsiella pneumoniae* (*n* = 28), *Escherichia coli* (*n* = 14), *Acinetobacter baumannii* (*n* = 13), and *Pseudomonas aeruginosa* (*n* = 11) (Figure 2C). They were also the most common bacteria detected by both mNGS and culture. The most common fungus detected by mNGS was *Aspergillus fumigatus* (*n* = 5), and the most common virus was *human herpesvirus 5* (*n* = 17). In addition, mNGS detected atypical pathogens, including *Mycobacterium tuberculosis complex*, *Coxiella burnetii*, and *Orientia tsutsugamushi*. The most common fungus detected by culture was *Candida glabrata* (*n* = 2). Conventional culture does not involve detection of the virus.

### Diagnostic performance of plasma mNGS in different patient taxa

**Figure 3.**
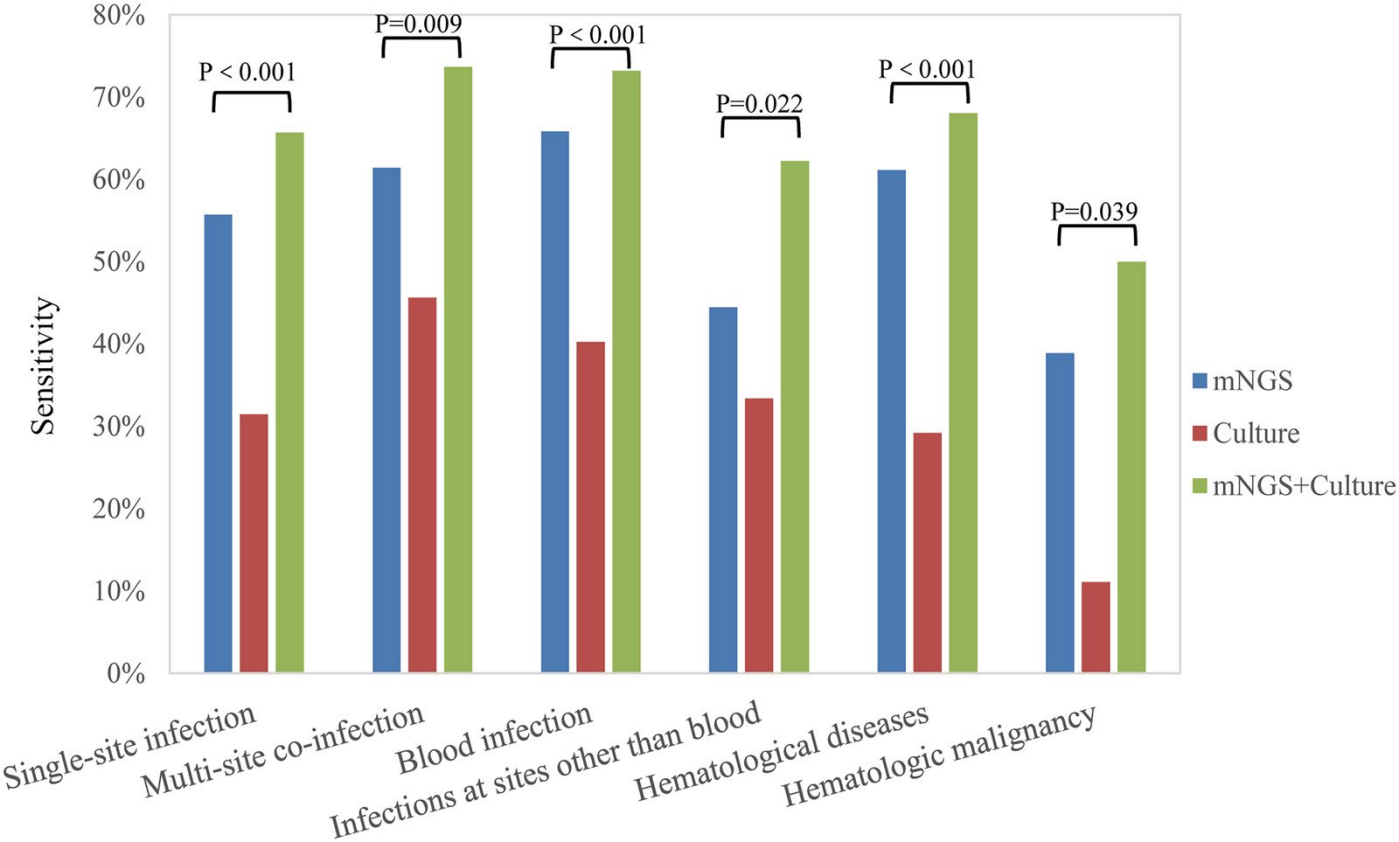
Detection sensitivity of mNGS method, culture method, and mNGS combined culture method in different groups.

Patient populations were divided according to infection site and type of infection. For the infection site, it was divided into single-site infection and multi-site co-infection. For the type of infection, it is divided into blood infections and infections from other parts of the blood. We also divided patients into groups of patients with haematological and patients with haematological malignancies. In all patient populations, there were significant differences in sensitivity between groups using mNGS method, cultures method, and mNGS combined cultures methods (Figure 3). Compared with culture methods, mNGS detection and mNGS combined culture methods showed higher sensitivity in all patient populations. Compared with single site infection, plasma mNGS and mNGS combined culture had higher detection sensitivity in patients with multi-site co-infection. Compared with other sites of infection, the mNGS combined culture method was more sensitive in patients with blood infection. Overall, plasma mNGS combined culture methods were more sensitive in patients with multi-site co-infections and blood infections.

### Effect of inflammatory markers on diagnostic performance of mNGS

**Figure 4.**
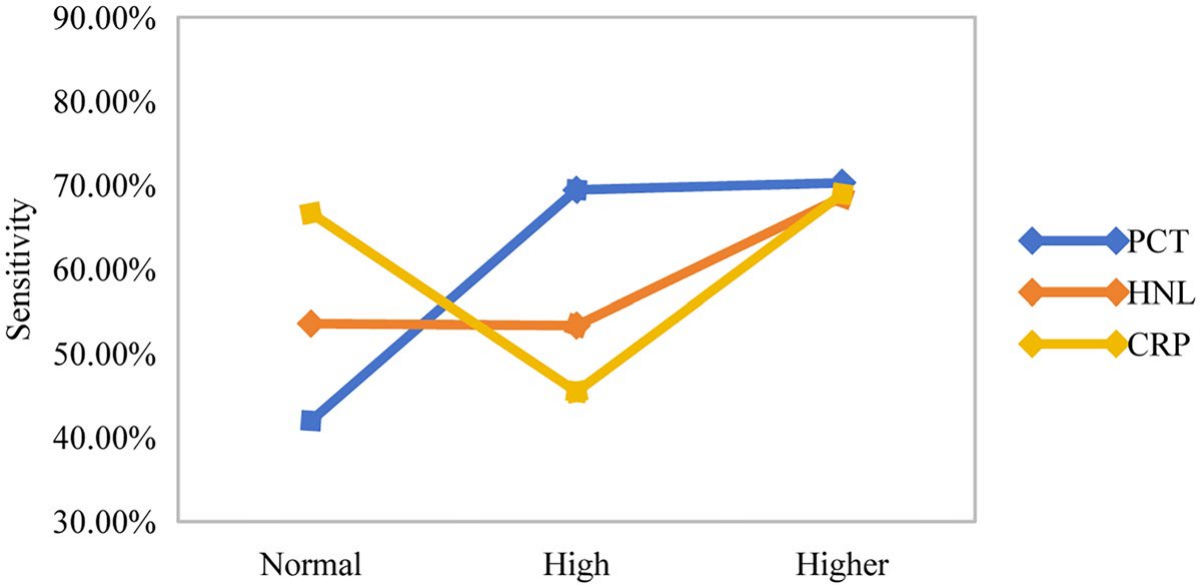
Detection sensitivity of mNGS in different inflammatory index ranges. The normal levels of PCT, HNL and CRP were <0.5 ng/mL, ≤ 115.55 ng/mL and ≤10 mg/L, respectively. The high levels of PCT, HNL and CRP were 0.5-10 ng/mL, 115.55-200 ng/mL and 10-99 mg/L, respectively. The higher levels of PCT, HNL and CRP were >10 ng/mL, >200 ng/mL and >100 mg/L, respectively.

PCT, HNL, and CRP were used as inflammatory markers for the diagnostic performance analysis of mNGS. When the PCT and HNL indices exceeds the normal threshold [25–28], plasma mNGS detection was more sensitive (Figure 4). The sensitivity of plasma mNGS increases with rising PCT and HNL levels, especially when the PCT level is >10 ng/mL or the HNL level is >200 ng/mL.

### Clinical effects of plasma mNGS on the treatment of infected patients

Among all patients with infectious diseases, plasma mNGS had a positive effect on the treatment of 54.33% of patients. 45.67% of patients were not adjusted for antibiotics according to mNGS. No negative effects were observed. In patients with no effect, untapered or de-escalated antibiotics were most common with a negative mNGS result. Of the 69 patients with a positive treatment effect, 45 underwent antibiotic adjustments, and in the remaining 24 patients, although no antibiotic adjustment was made, a positive result contributed to the fact that the pathogen had been covered by empiric therapy (Table 3). We further explored the practical clinical impact value of mNGS by examining changes in the length of hospital stay (LOHS) and the interval from the mNGS report time to discharge. However, no significant differences were observed in these two indicators between the positive impact group and the no-impact group 30.43 ± 29.72 vs. 29.69 ± 41.97 days (*p* = 0.908), and 19.84 ± 23.22 vs. 22.68 ± 41.27 days (*p* = 0.629).

**Table 3.** Clinical impact of plasma cfDNA on the treatment of infected patients.

Clinical impact	Grade	Description	Case number, n (%)
Positive	M1	Initialization of the appropriate antibiotics treatment	20 (15.75%)
	M2	Antibiotic escalation	22 (17.325)
	M3	Antibiotic de-escalation	3 (2.36%)
	M4	Confirmed empirical treatment	24 (18.90%)
No effect	M5	No adjustment in treatment while the result was positive	13 (10.24%)
	M6	The patient was discharged or dead	1 (0.79%)
	M7	No adjustment in treatment while the result was negative	42 (33.07%)
Negative	M8	NGS led to unnecessary treatment	–

## Discussion

In our study, we systematically compared plasma mNGS and culture assays in pairs. The total positive rate of plasma mNGS was significantly higher than that of the culture method (55.71% vs 25.10%, *p* < 0.001), and the positive rate of blood culture was only 12.86% (*p* < 0.001). However, unlike other studies, we had a lower rate of plasma mNGS positivity in our study, which may be due to the fact that we enrolled patients with suspected infection, and not all of the patients had blood at the site of infection [3,29]. Our study found that when the clinical diagnosis of infectious diseases was used as the criterion, the sensitivity of plasma mNGS was 58.27%, which was higher than that of conventional cultures (*p* = 0.089), but when plasma mNGS was combined with culture, the sensitivity for detecting infectious diseases increased to 69.29%. A negative result for mNGS does not rule out pre-existing infection, and the concentration of cfDNA varies depending on the degree of infectious disease, so when mNGS is combined with culture, it helps confirm the clinical diagnosis. Although cfDNA mNGS has proven to be advantageous in pathogen detection, our findings suggest that mNGS should not be used as a replacement for cultures, but rather as a complement to conventional microbial detection methods for more accurate pathogen identification.

In this small study of 140 suspected infected patients, the most commonly detected bacteria in plasma mNGS and cultures were *Klebsiella pneumoniae*, *Escherichia coli*, *Acinetobacter baumannii*, and *Pseudomonas aeruginosa*. This was also consistent with the results of previous studies [7,30,31]. In our study, the lungs and blood were the most common sites of infection, and *Klebsiella pneumoniae*, *Escherichia coli*, *Acinetobacter baumannii*, and *Pseudomonas aeruginosa* were also common pathogens of lung infections and bloodstream infections [32]. mNGS was more sensitive in the detection of fungi than culture, and the most common fungus detected was *Aspergillus*, which is also a common fungal pathogen in bloodstream infections [32–34]. In addition, the incidence of atypical pathogen infections is increasing rapidly, and accurate diagnosis can improve patient outcomes. In this study, mNGS also detected *Mycobacterium tuberculosis complex*, *Coxiella burnetii*, and *Orientia tsutsugamushi*. Compared with the detection of atypical pathogens, culture methods are not sensitive.

In the current clinical diagnosis of patients with suspected infection, blood cultures are usually performed to identify the organism, but the positive rate of blood cultures is often low. This is especially true in patients with infections at other sites. So far, plasma mNGS has been mainly used in the study of bloodstream infections [12,35–37]. However, there are very few studies on which groups of patients plasma mNGS is more appropriate. Similar to previous studies, plasma mNGS was more sensitive in detecting bloodstream infections in patients, with a sensitivity of 73.17% when plasma mNGS was combined with culture [37]. Plasma mNGS is more sensitive in patients with multi-site infection than in patients with single site infection, and may be due to the release of nucleic acids from the lesion site into the circulatory system, which is more easily detected by mNGS. It is important to note that in this study, the final clinical diagnoses—based on a comprehensive evaluation of patients’ clinical manifestations, imaging findings, laboratory tests, mNGS-based etiological detection results, and therapeutic responses—were used as the reference standard for assessing the clinical impact of mNGS. However, this approach may introduce potential biases. Since the final diagnosis relies heavily on clinicians’ empirical judgment, cases presenting with atypical symptoms or lacking definitive etiological evidence (e.g. culture-negative infections) may be subject to inter-observer variability, thereby compromising the objectivity of the diagnostic reference standard. Moreover, incorporating mNGS results into the final diagnostic criteria may create a circular reasoning problem—evaluating mNGS performance using a standard that already includes mNGS data—potentially leading to an overestimation of its diagnostic accuracy and obscuring its true independent diagnostic value. Additionally, while the final clinical diagnosis determines the presence of infection based on clinical context, mNGS may detect colonizing organisms rather than active pathogens. In such instances, if the clinical diagnosis excludes infection, mNGS-positive results may be misclassified as false positives, thereby underestimating specificity. Conversely, mNGS may fail to detect low-abundance pathogens due to technical limitations, potentially leading to underestimation of sensitivity. The implementation of a third-party blinded adjudication process could help mitigate these biases. Nonetheless, the optimal methodology for accurately and objectively evaluating the diagnostic performance of mNGS remains a subject requiring further in-depth investigation.

In this study, the specificity of mNGS is 76.92%, which means that nearly a quarter of non-infected patients will have positive mNGS results, i.e. false-positive results. False positives may lead to overtreatment, waste of medical resources, interference with clinical judgment, and delay in the diagnosis and treatment of non-infectious diseases. The causes of false-positive mNGS results include microorganisms or nucleic acids introduced during sample collection or transportation, normal human colonizing microorganisms, background microorganisms in the detection process, cross-contamination between samples, and incorrect species identification [38–41]. With technological advancements, the impact of these factors has been decreasing, but they are difficult to avoid. It is worth noting that the pathogenic microorganisms detected by mNGS are the ones actually present in the samples, but not all of them are necessarily pathogenic. To determine whether they are pathogenic microorganisms, it is necessary to improve the accuracy and sensitivity of mNGS detection, reduce the false-negative rate of mNGS reports to rule out infections, and at the same time minimize contamination to avoid the interference of false positives on clinical judgment. Therefore, to minimize the occurrence of false-positive results, strict aseptic operation is required, and samples should be collected from lesion sites and sent for inspection immediately. A complete and high-quality pathogenic microorganism database should be constructed and regularly updated. A database of reagent and environmental background nucleic acids should be established to filter contaminated sequences and distinguish between background pathogens and pathogenic pathogens. Full-process quality control should be implemented: negative and positive controls should be set for each batch of experiments, internal standards should be added to samples, quality control points should be set in nucleic acid extraction and library construction links, and comprehensive quality control should be performed on the off-machine data. The microbial genomic information obtained by mNGS detection can not only be used for pathogen identification, but also for further analysis of drug-resistant genes [42], which could provide important references for clinical practice. However, it has limitations: drug-resistant genes only account for a small part of the entire genome of pathogenic microorganisms, resulting in limited detection sensitivity and high false-negative rate, and it cannot fully reflect the actual drug-resistant phenotype. Affected by factors such as sample type and quality, when multiple microorganisms are detected, it is difficult to accurately match drug-resistant genes to specific microorganisms [43]. Therefore, in clinical judgment of drug resistance, routine bacterial culture and drug sensitivity testing still need to be carried out. Combined with risk factors such as the patient’s antimicrobial exposure history, living environment, underlying diseases, and the degree of medical care dependence, multiple detection technologies should be used for cross-validation to clarify bacterial drug resistance.

Infected patients cause an inflammatory response by releasing cytokines and inflammatory mediators [44]. CRP has been identified as an important risk factor for positive blood cultures in patients with bloodstream infections [45]. Previous studies have also found that CRP may lead to mNGS-positive results in patients with acute infection [46]. In our study, the sensitivity of plasma mNGS increased with increasing PCT and HNL indexes. Yan et al. also found a high correlation between the abundance of microorganisms in the blood and PCT levels [47].

In addition, we assessed the real-world clinical impact of plasma mNGS assays on the treatment of infected patients. In our study, 69 (54.33%) of the 127 patients who were eventually clinically diagnosed with infection had a positive impact on clinical treatment based on mNGS results. In a previous study of infectious diseases, 82 karius tests were evaluated from 39 adults and 43 children. The positivity rate was 61.0% (50/82), but plasma mNGS only had a positive effect on 7.3% of patients [48]. In another study of infections in neutropenia and non-neutropenia patients with hematologic diseases, plasma mNGS had a positive effect on 38.62% of patients [24]. This difference may be influenced by variations in patients’ baseline characteristics, timing of testing, antibiotic management policies, clinical medication habits, and definitions of study endpoints. The median ages of patients in the above two studies and this study were 25.2, 39, and 58 years, respectively. The proportions of immunocompromised patients were 64.6%, >53%, and 20%, respectively. The proportions of patients who had already used antibiotics when mNGS testing was performed were unknown, 87.6%, and 49.6%, respectively. The clinical outcome was whether the mNGS results guided adjustments to clinical medication. From the above data, it can be seen that older age, a lower proportion of immunocompromised patients, and a lower proportion of patients who had already used antibiotics when mNGS testing was performed may be important reasons for the higher proportion of positive clinical impacts in this study.

Finally, there are some limitations to this study. The clinical diagnosis of infectious diseases in this study is based on a comprehensive approach, including microbiological testing, clinical manifestations, imaging results, and results after anti-infective therapy. Confirmatory testing was not performed for all microorganisms detected in the sample, which may affect the definition of detected microorganisms. In addition, not all patients had mNGS testing of plasma before antibiotic testing. Previous empiric antimicrobial therapy may also have some impact on the detection of mNGS. Thirdly, more appropriate clinical outcome indicators need to be added to further clarify the practical application value of mNGS in treatment.

## Conclusions

This study highlights the significant potential of plasma cfDNA mNGS for the detection of pathogens in suspected infected patients. When cultured in combination with plasma mNGS testing, the detection rate of pathogens can be significantly improved, especially in patients with multisite infections and bloodstream infections. Plasma mNGS has higher detection sensitivity when PCT > 5 ng/mL or HNL > 200 ng/ml in patients with suspected infections. In addition, plasma mNGS can help optimize antibiotic management in infected patients. Moreover, clinicians need to be vigilant that due to the risk of false positives, if the test results are not interpreted properly, mNGS may instead lead to overtreatment.

## Data Availability

The data presented in the study are deposited in the SRA (https://www.ncbi.nlm.nih.gov/sra/) repository, accession number PRJNA1180703.
